# Primary leiomyosarcoma of thyroid gland: the youngest case

**DOI:** 10.11604/pamj.2017.26.113.11472

**Published:** 2017-03-01

**Authors:** Mouna Ayadi, Azza Gabsi, Khdija Meddeb, Amina Mokrani, Yosra Yahiaoui, Feryel Letaief, Nesrine Chraiet, Henda Rais, Amel Mezlini

**Affiliations:** 1 Service d’Oncologie Médicale, Institut Salah Azaiez Tunis, Faculté de Médecine de Tunis, Université el Manar, Tunisie

**Keywords:** Thyroid, sarcoma, pathology, treatment

## Abstract

Primary leiomyosarcomas of the thyroid gland are extremely rare. We report a case of a 32 year-old women with a multinodular goiter. She underwent total thyroidectomy. The tumor histology showed spindle-shaped cells that expressed desmine, caldesmone and smooth muscle actine but were negative cytokeratins.

## Introduction

Leiomyosarcoma constitutes 6% of head and neck tumors and they are exceedingly rare in thyroid gland itself [1]. It is believed that primary leiomyosarcoma of thyroid gland (PLT) originate from the smooth muscles of blood vessels in thyroid gland which has abundant vascularization. To the best our knowledge, there are only 22 well-documented PLT cases in literature.

## Patient and observation

**Figure 1:**
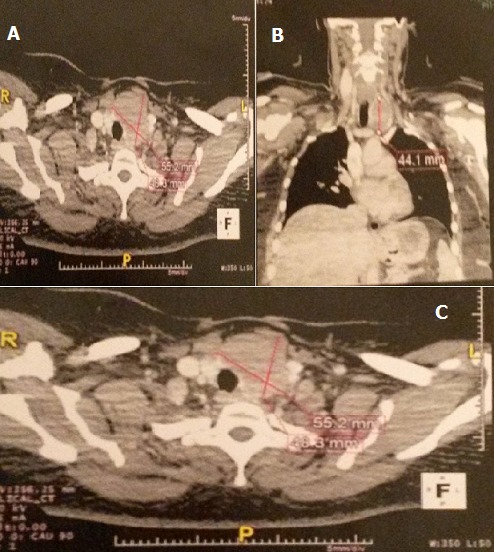
Computed tomography showed a thyroid nodule of the left lobe extended to the isthmus and the right lobe with anterior and posterior capsular rupture contracting close contact with the vascular axis left carotid-jugular plunging into the cervicothoracic away from the hole aortic arch (A,B,C)

We present a case of a 32-year-old woman with a history of gradual enlargement of the anterior neck. The medical history was unremarkable and no comorbidities existed. There was no history of radiation exposure. Clinical examination revealed a multinodular goiter. Computed tomography showed a thyroid nodule of the left lobe extended to the isthmus and the right lobe with anterior and posterior capsular rupture contracting close contact with the vascular axis left carotid-jugular plunging into the cervicothoracic away from the hole aortic arch (Figure 1). There was no evidence of lung lesions. The patient underwent a surgical exploration. There was a hard left lobe nodule of 5 cm infiltrating the adjacent muscles and partially infiltrates the trachea. The intraoperative consultation pathology diagnosis was: undifferentiated carcinoma. A total thyroidectomy was realized. Histological examination showed a proliferation of elongated spindle-shaped cells, arranged in interweaving fascicles of varying sizes, intersected at right angles. Tumor cells are atypical with strange nuclei. Chromatin is distributed inhomogeneously. The cytoplasmic membrane is irregular and thick. The nucleolus is very large. The tumor realize a pushing against thyroid parenchyma which is separated with a fibrous capsule.The mitotic rate was extremely high (19 mitosis/10 high power field), and atypical mitotic figures were also present. The neoplasia showed invasion of the peri-glandular fat tissue. Immuno-histochemical staining of the slides with caldesmon, desmin, PanCK, CK5-6, CK7, myogenin, epithelial membrane antigen (EMA), CEA, thyroid transcription factor (TTF-1), pancytokeratin, smooth muscle actin (SMA), MelanA, S 100 protein, CD 45, CD3, CD30, CD 20, CD 15, CD34, ALK, calcitonin and KI 67 protein was performed. The tumour was strongly positive for caldesmon, SMA, desmin, and negative for pancytokeratin and other epithelial, lymphoid and melanocytic markers. On the basis of the clinical, radiographic, histopathological and immunohistochemical features, the final diagnosis was primary thyroid leiomyosarcoma, FNCLCC grade 3. In multidisciplinary tumour board, it was decided that adjuvant loco regional RT and chemotherapy by ifosfamide and doxorubicin.

## Discussion

Primary leiomyosarcoma of the thyroid gland (PLT) is exceedingly rare and accounts for 0.014% of thyroid tumors. It occurred in older patients with of age 67 years without sex prevalence. Our patient is the youngest women described in literature. The etiology of PLT is not clear yet. Most of the reports in the literature show that cases are not associated with a benign or malign pre-existing thyroid lesion, and there is not any history of radiation exposure [2]. The PLT usually goes along with fast growing, painless masses and the symptoms of hoarseness and dysphagia [3, 4]. PLT has no characteristic imaging features that might be useful for diagnostic purposes. In thyroid isotope scanning, thyroid leiomyosarcoma can demonstrate a cold nodule or hyperplasia with increased and decreased uptake of radioactive iodine [1]. Ultrasound can reveal an ill-defined or well-defined hypo-echogenic mass with cystic or calcified components [1]. But these modalities can be very helpful to assess for local aggressiveness including extra capsular extension and invasion to the airway and esophagus. CT imaging of PLT in most of the thyroid cancer show a low-density mass with calcification and necrosis [1]. The tumor was commonly delineates an isointense mass on T1-weighted MR images and a mass effect of intermediate signal on T2-weighted images. After gadolinium injection, the lesion generally demonstrates a fair enhancement on T1-weighted images [5]. Grossly, PLT are large fleshy white-gray masses, with foci of fresh tumor necrosis and hemorrhage, and a tendency for cystic degeneration. Microscopically, the pattern of growth is usually fascicular, with tumor bundles intersecting each other. Certain tumors also present areas with a whorled appearance. The individual neoplastic cells are elongated, with abundant acidophilic fibrillary cytoplasm; the nucleus is generally centrally located and typically blunt-ended or ‘cigar-shaped’. These features also appear on cytological samples. The degree of nuclear atypia is highly variable and the mitotic activity varies considerably. High mitotic activity is virtually diagnostic of malignancy, although a PLT must be strongly suspected for a tumor that is widely necrotic, hemorrhagic and with significant atypia, even if the mitotic index is low. Immunohistochemically, thyroid leimyosarcoma show reactivity for vimentin, smooth muscle actin, muscle-specific actin, smooth muscle myosin, desmin, H-caldesmon and basal lamina components, including laminin and type IV collagen. H-caldesmon is a muscle marker used to discriminate between smooth muscle cells and myofibroblasts; this marker appears to be associated with the degree of differentiation. Other antigens sporadically identified in thyroid leiomyosarcoma are S-100 protein, estrogen and progesterone receptor proteins, raising the possibility of hormonal responsiveness [6].

The main differential pathologic diagnosis includes undifferenciated thyroid carcinoma, solitary fibrous tumors, spindle cell tumors with thymus like differentiation, medullary carcinoma and other sarcomas [1]. In our case, the diagnosis of PLT was made on the pathological and immunohistochemical features of the tumor, which were similar to those found in literature. To date, it is not clear whether therapy is effective in prolonging survival, as demonstrated in 22 reported cases [1]. Rapid locoregional infiltration and diffuse brain or lung metastases are responsible for the high mortality rate. Total or near-total thyroidectomy, for the majority of thyroid pathologies, associated with therapeutic modified radical neck dissection should be considered for intrathyroidal disease [7–11]. Chemotherapy has not shown any therapeutic efficacy. Wang et al and Raspollini et al reported interesting data in the management of thyroid and uterus leiomyosarcomas through the overexpression of c-Kit proto-oncogene, a tyrosine kinase receptor [4, 12]. However, the use of imatinib mesylate (tyrosine kinase inhibitor) did not prevent the relapse and the fatal outcome in one patient with PLT associated with lung metastases [13]. In the case of locoregional infiltrating disease, surgery may be performed to prevent airway or esophageal obstruction. Often, therapies do not produce any clinical benefit, only palliative results.

## Conclusion

PLT remains a fatal tumor, invariably associated with a dismal prognosis, and, although notable improvements in oncology, an efficacious multimodal treatment protocol is lacking. To modify the poor surgical outcomes, novel and effective adjuvant therapeutic strategies, based on a molecular approach, are required.
